# Global Metabolomic Analyses of the Hemolymph and Brain during the Initiation, Maintenance, and Termination of Pupal Diapause in the Cotton Bollworm, *Helicoverpa armigera*

**DOI:** 10.1371/journal.pone.0099948

**Published:** 2014-06-13

**Authors:** Yu-Xuan Lu, Qi Zhang, Wei-Hua Xu

**Affiliations:** State Key Laboratory of Biocontrol, School of Life Sciences, Sun Yat-Sen University, Guangzhou, China; University of Cincinnati, United States of America

## Abstract

A strategy known as diapause (developmental arrest) has evolved in insects to increase their survival rate under harsh environmental conditions. Diapause causes a dramatic reduction in the metabolic rate and drastically extends lifespan. However, little is known about the mechanisms underlying the metabolic changes involved. Using gas chromatography-mass spectrometry, we compared the changes in the metabolite levels in the brain and hemolymph of nondiapause- and diapause-destined cotton bollworm, *Helicoverpa armigera*, during the initiation, maintenance, and termination of pupal diapause. A total of 55 metabolites in the hemolymph and 52 metabolites in the brain were detected. Of these metabolites, 21 and 12 metabolite levels were altered in the diapause pupal hemolymph and brain, respectively. During diapause initiation and maintenance, the number of metabolites with increased levels in the hemolymph of the diapausing pupae is far greater than the number in the nondiapause pupae. These increased metabolites function as an energy source, metabolic intermediates, and cryoprotectants. The number of metabolites with decreased levels in the brain of diapausing pupae is far greater than the number in the nondiapause pupae. Low metabolite levels are likely to directly or indirectly repress the brain metabolic activity. During diapause termination, most of the metabolite levels in the hemolymph of the diapausing pupae rapidly decrease because they function as energy and metabolic sources that promote pupa-adult development. In conclusion, the metabolites with altered levels in the hemolymph and brain serve as energy and metabolic resources and help to maintain a low brain metabolic activity during diapause.

## Introduction

Most insect species have evolved an adaptive strategy known as diapause (developmental arrest) to survive an unfavorable season [1], [2]. Diapause is typically characterized by reduced metabolic activity and enhanced stress tolerance and is induced by environmental signals (day length, temperature, etc.). The neuroendocrine system translates the physical signals into hormonal factors that induce the individual to enter diapause. For example, in the cotton bollworm, *Helicoverpa armigera* that is an important agricultural noctuid moth in the world, its pupal diapause is induced by incubating larvae under short day-light and low-temperature conditions that down-regulate the expression of neuropeptide prothoracicotropic hormone (PTTH) in the pupal brain. The low PTTH expression level inhibits the prothoracic glands from synthesizing steroid hormone ecdysteroids (or ecdysone), which are needed to promote continuous development [3]. As a result, the pupa enters overwintering diapause. In the laboratory condition, *H. armigera* pupal diapause is initiated by exposing 5th–6th instar larvae under short day (10 h light: 14 h dark) and 20 °C conditions, and the lifespan is over three times than in their nondiapause counterparts reared under long day (14 h light: 10 h dark) and 25 °C without diapause.

The insect gradually reduce its metabolic activity during diapause initiation, and maintain the low metabolic activity during diapause maintenance for overwintering; when the environment is suitable, the insect physiological state will be reached in which direct development may activate [3], [4]. Until recently, a few metabolite (e.g. glycogen, glycerol, sorbitol, and trehalose) changes that occur in diapause individuals compared with nondiapause individuals have been widely investigated, sorbitol and trehalose have been demonstrated predominant in diapausing pupae for energy resources and cryoprotectants in *H. armigera* [5]. In the egg diapause species *Bombyx mori*, glycogen is converted to sorbitol and glycerol during diapause [6], [7], and oxygen consumption and sorbitol utilization increase when diapause is terminated [8]. In addition, lipid and glycerol levels are higher at the onset of pupal diapause and the glycogen level is lower during diapause in the flesh fly *Sarcophaga crassipalpis* [9]. Similarly, a reduction in glycogen and an increase in glycerol have also been observed in overwintering rice stem borer, *Chilo suppressalis*, larvae [10]. These metabolites, such as glycerol, sorbitol and trehalose, can act as antifreezes and may increase an insect's survival rate during winter [11], [12]. Moreover, sorbitol and trehalose not only act as antifreezes but also act as arresters during *B. mori* embryonic diapause [13]. Thus, metabolites are involved in many biological processes during insect diapause.

Previous studies have primarily focused on a few metabolites, and little is known about the other metabolites that are involved in diapause. Metabolomics is an emerging field that it allows a more global picture of metabolic changes underpinning the phenotype of interest in biological systems and offers a broad and bias-free view of the biological processes involved [14]. Moreover, metabolomics is the downstream result of gene and protein expression and links the genotypes and phenotypes in the organism [15]. Therefore, a metabolomic approach has been used to study insect diapause in *S. crassipalpis*, and many metabolite levels were observed to be altered in the diapause individuals using gas chromatography-mass spectrometry (GC-MS) [16].

Insects have an open body cavity, and all of the organs and tissues are submerged in hemolymph, including the brain. Hemolymph transports substrates for development and diapause. Thus, the metabolic activity of the brain (brain activity) depends on the metabolites in the hemolymph. To provide clues about diapause mechanisms why there is low metabolic brain activity in diapausing individuals, we performed a GC-MS analysis to compare the metabolomic changes in the pupal hemolymph and brains of nondiapause and diapause *H. armigera* individuals during the initiation, maintenance, and termination of pupal diapause. A total of 55 metabolites in the hemolymph and 52 metabolites in the brain were detected, and these metabolites play various roles in insect diapause. The accumulation of proline and serine in hemolymph may serve as energy source during diapauses, the phosphoric acid levels affect phosphorylated protein levels in the brain, and a lot of metabolite levels decrease in hemolymph for resuming development during diapause termination.

## Materials and Methods

### Insect rearing

*H. armigera* larvae were kindly provided by Dr. Jian-Ya Su, Nanjing Agricultural University (Nanjing, China). When the larvae were reared on an artificial diet and under long day conditions at 20 °C (L14: D10), all of the pupae developed without entering diapause. When the larvae were reared at 20 °C and under short day conditions (L10: D14), more than 90% of the individuals entered pupal diapause. The pupal samples collected on day 0, 1, 2, 3, 4, and 10 after pupation represent diapause initiation, and the samples collected on day 21 after pupation represent diapause maintenance. To investigate diapause termination, 1 µg 20-hydroxyecdysone (20E) was injected into diapausing pupae (day 21 after pupation), and the samples were collected at 24, 48, and 72 h after injection. Pupal brains were dissected in 0.75% NaCl medium and stored at −80°C. A sample consisted of 30 brains, and 4 replicates of these samples were prepared. The hemolymph (100 µL) was collected from 10 pupae and combined as a single sample, and 4 replicates of these samples were prepared. The samples were centrifuged at 12000 g for 5 min to remove the hemocytes, and the supernatant was transferred to a new 1.5 mL tube and stored at −80°C.

### Sample preparation and derivatization

For the hemolymph metabolomic analysis, 100 µL distilled water containing 0.1 mg/mL sucrose (internal standard) and 800 µL methanol were added to each sample (100 µL hemolymph). Next, the sample was vortexed for 10 s, incubated on ice for 1 h, and centrifuged at 20000 g for 10 min. Then, 600 µL supernatant was transferred to a GC vial and dried using a vacuum for approximately 3 h. Next, 90 µL freshly prepared methoxylamine hydrochloride (15 mg/mL in pyridine; Sigma) was added to the GC vial, and the sample incubated for 16 h at room temperature. The sample was then added to 90 µL MSTFA (containing 1% TMCS; Sigma) for trimethylsilylation for 1 h. Finally, the derivatization reaction was stopped by adding 120 µL hexane to the sample.

For the metabolomic analysis of the brain, the samples were derivatized following the method previously described by Zhang *et al*. [17]. Briefly, each sample, consisting of 30 brains, was homogenized in a 600 µL methanol-chloroform mixture (2∶1) and then sonicated for 15 min. Next, 200 µL distilled water containing 0.01 mg/mL sucrose (internal standard) and 200 µL chloroform were added to the sample, and the sample was centrifuged at 20000 g for 10 min. The aqueous layer was then transferred to a GC vial and dried using a vacuum for 2–3 h. Next, 10 µL freshly prepared methoxylamine hydrochloride (15 mg/mL in pyridine; Sigma) was added to the GC vial, and the sample incubated for 16 h at room temperature. The sample was added to 10 µL MSTFA (containing 1% TMCS; Sigma) for trimethylsilylation for 1 h. Finally, the derivatization reaction was stopped by adding 20 µL hexane to the sample.

### GC-MS analysis

A total of 1 µL derivatization sample was auto-injected into the 7890A/5975C GC-MS (Agilent). The split ratio was 1∶50 for the hemolymph sample and 1∶10 for the brain sample. For the metabolomic analysis, a HP-5 ms column (length 30 m, I.D. 25 mm; Agilent) was used. The injector temperature was maintained at 280 °C. The initial oven temperature was set at 50 °C. The oven temperature increased 5 °C/min to 300 °C and was then maintained at 300 °C for 5 min. Data were collected from the full scan mode across a mass range of 50 to 450 m/z.

### Data analysis

The individual peaks were identified by comparing the retention times with the metabolite standards. Other peaks were identified by matching the mass spectra against the National Institute of Standards and Technology (NIST). RSI value over 700 was considered a good match. The overlapping peaks were deconvoluted using traces for single ions. The individual integrated peak areas were converted to a relative response ratio relative to the internal standard (sucrose) peak area. One-way ANOVA analysis was used to analyze all of the metabolites, and Benjamini-Hochberg method was used to correct the p-value. A p-value<0.05 was considered a significant difference. For the principal component analysis (PCA), the relative response ratios for all of the metabolites were analyzed using MetaGeneAlyse 1.7 (Max Planck Institute of Molecular Plant Physiology, Germany) and separated by their principal components.

## Results

### General metabolomic profiles

A GC-MS analysis was performed to detect metabolites in the brains and hemolymph of *H. armigera* diapause- and nondiapause-destined pupae during diapause initiation, maintenance, and termination. A total of 55 metabolites were identified in the hemolymph, consisting of 16 amino acids, six sugar and polyols, six metabolic intermediates, three small molecules and 24 other metabolites (Table 1). Meanwhile, 52 metabolites were detected in the pupal brain, consisting of 23 amino acids, 11 sugar and polyols, five metabolic intermediates, one small molecule and 12 other metabolites (Table 2). A total of 75 different metabolites were identified in the pupal brain and hemolymph: 32 of the metabolites identified were found in the hemolymph and brain, 23 metabolites were hemolymph specific, and 20 metabolites were brain specific.

**Table 1:** Metabolites identified from the hemolymph of *H. armigera*.

Amino acids	Sugars and polyols	Metabolic intermediates	Small molecules	Other metabolites
(16)	(6)	(6)	(3)	(24)
Alanine	* Arabinopyranose	* *α*-ketoglutanic acid	* Free amine	1.4-butanediamine
*β*-alanine	* Glucose*	* Fumaric acid*	* Free phosphate	* 1.2.3.-5-methylthiadiazole
Glutamate	Myo-inositol	* Isocitric acid*	Phosphoric acid	* 2-aminoethyl dihydrogen phosphate
Glycine	* Rhamnofuranose	* Malic acid*		* 2-hydroxypentanedioic acid
Isoleucine	* Turanose	* Pyruvate*		* 3-aminoisobutyric acid
Leucine	*Trehalose*	* Succinic acid*		3-hydroxypropanoic acid
Lysine				* 3-hydroxy-3-methylpentanedioic acid
Methionine				* 3.6-dihydroxyhexanoic acid
Ornithine				* Butanoic acid
Phenylalanine				* Cholesterol
Proline				D-gluconic acid
Serine				* Ethyl-2-hydroxy-2-(2-hydroxyphenyl)acetate
Threonine				*γ*-aminobutyric acid (GABA)
Tryptophan				* Linoleic acid
Tyrosine				* Methylcitric acid
Valine				* N-acetylglucosamine
				Octadecenoic acid
				Pantothenic acid
				* Palmitic acid
				* Ribonic acid
				* Sitosterol
				* Stearic acid
				Urea
				* Uridine

*The metabolite is only found in hemolymph. The metabolites that had been reported in reference 19 are italicized.

**Table 2:** Metabolites identified from the brain of *H. armigera*.

Amino acids	Sugars and polyols	Metabolic intermediates	Small molecules	Other metabolites
(23)	(11)	(5)	(1)	(12)
Alanine	* 1.3-propanediol	* Fumaric acid*	Phosphoric acid	1.4-butanediamine
* Asparagine	* Fructose	* Isocitric acid*		3-hydroxypropanoic acid
* Aspartate	* Galactose	* Malic acid*		* 4-phenylbutyric acid
* β*-alanine	* Glucose*	* Pyruvate*		* *α*-hydroxyglutaric acid
* Cystine	* *Glucose-6-phosphate*	* Succinic acid*		D-gluconic acid
Glutamate	* Glycerol			*γ*-aminobutyric acid (GABA)
* Glutamine	Myo-inositol			* Hexadecanoic acid
Glycine	* Mannose-6-phosphate			* Isoxanthopterin
* Homocysteine	* Ribose			Octadecenoic acid
Isoleucine	* Sorbitol			Pantothenic acid
Leucine	* Trehalose*			* Ribitol
Lysine				Urea
Methionine				
* *N*-Acetylaspartate				
* *N*-acetylglutamate				
Ornithine				
Phenylalanine				
Proline				
Serine				
Threonine				
Tryptophan				
Tyrosine				
Valine				

*The metabolite is only found in brain. The metabolites that had been reported in reference 19 are italicized.

### Changes in metabolite levels during diapause initiation

**Figure 1:**
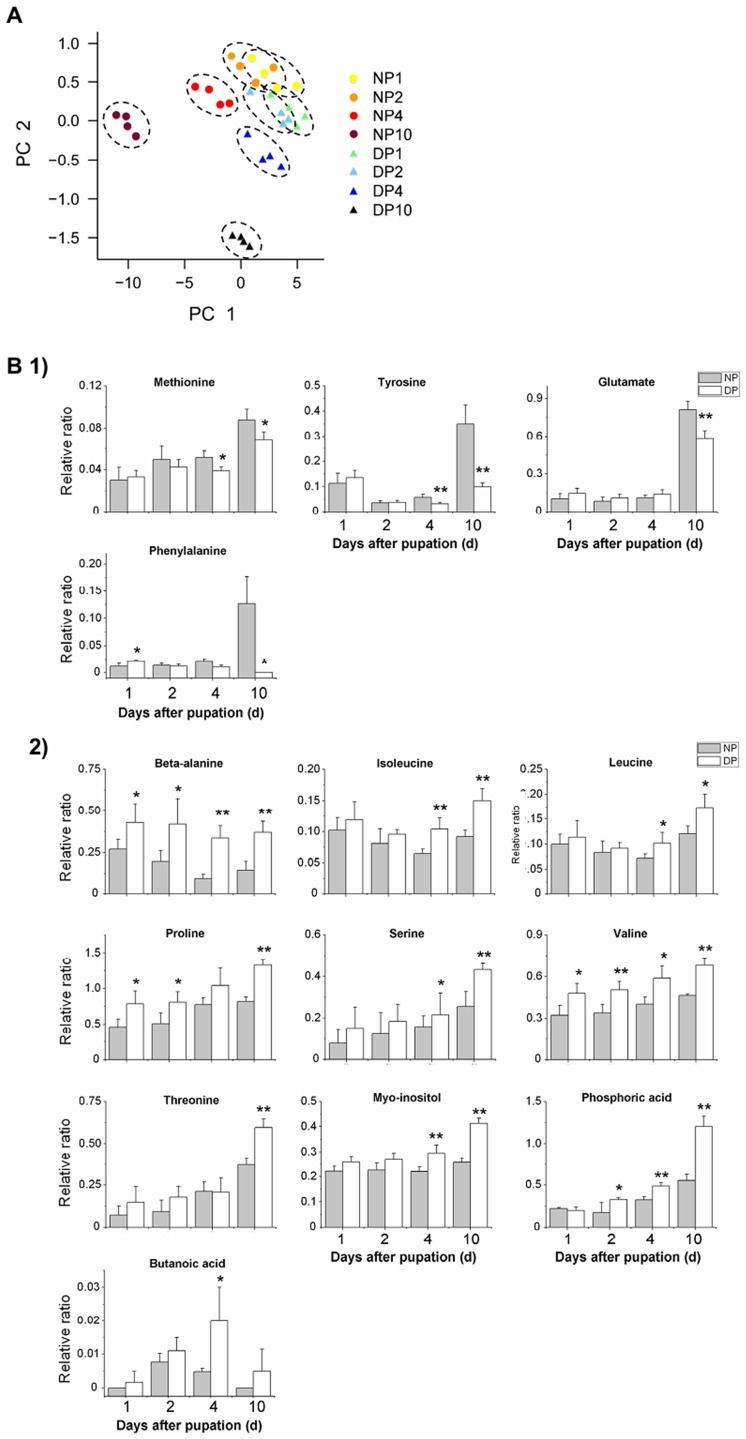
Principal component analysis (PCA) score plots and altered metabolites levels in the hemolymph. (A) PCA and (B) altered metabolites levels in the hemolymph of nondiapause- and diapause-destined pupae. The first principal component (PC1) containing valine, threonine, methionine, and phosphoric acid, etc. accounted for 44.0% of total variation, and second principal component (PC2) containing glycine, *β*-alanine, phenylalanine, and D-gluconic acid accounted for 18.9% of total variation in hemolymph PCA plot. The metabolites were detected in day 1 to 10 pupae. 1) and 2) represent reduced- and elevated-metabolite levels, respectively, in diapause-destined pupal hemolymph. The samples within a treatment group were circumscribed spatially by the dashed line. The relative ratio is the ratio of metabolite peak area to internal standard (sucrose) peak area. All bars represent the mean ± S.D. from four repeats. *, *p*<0.05; **, *p*<0.01 (determined by one-way ANOVA). NP, nondiapause pupa; DP, diapause-destined pupa. Arabic number (1, 2, 4, and 10) represent the days after pupation.

**Figure 2:**
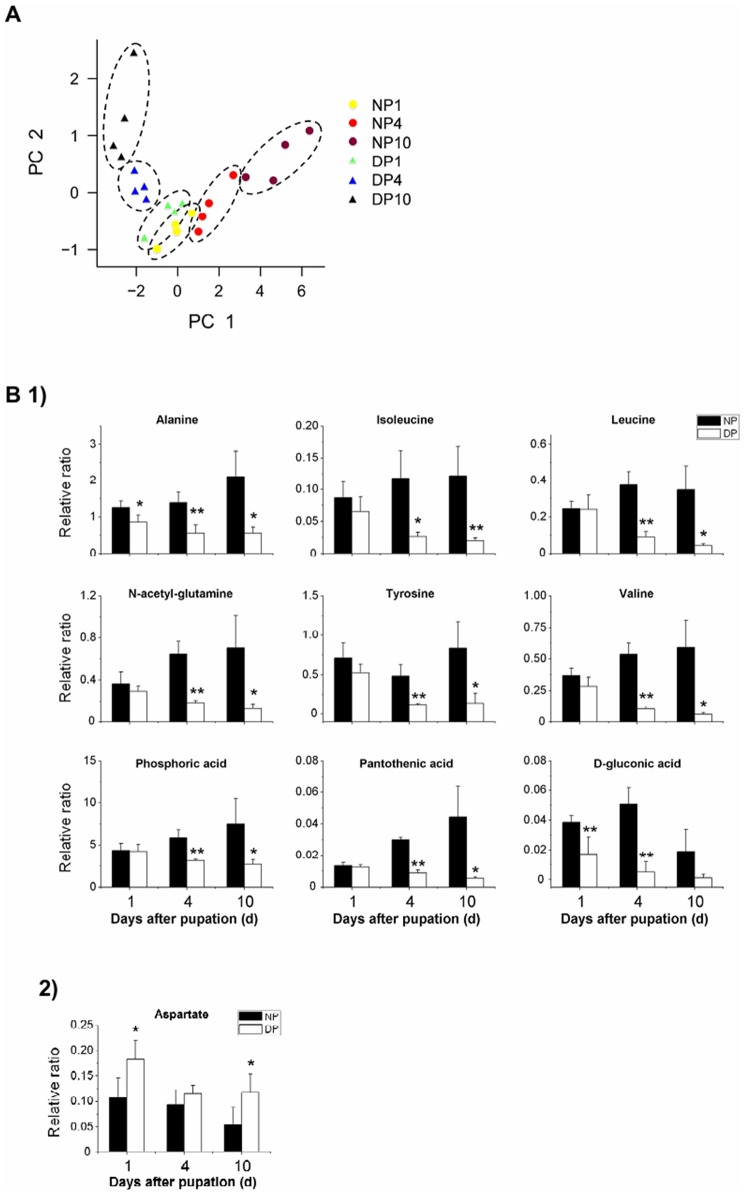
Principal component analysis (PCA) score plots and altered metabolites levels in the brain. (A) PCA and (B) altered metabolites levels in the brain of nondiapause- and diapause-destined pupae. The metabolites were detected in day 1 to 10 pupae. PC1 containing valine, alanine, leucine, isoleucine threonine, methionine, and phosphoric acid, etc. accounted for 52.2% of total variation, and PC2 containing *β*-alanine and tryptophan accounted for 11.1% of total variation in brain PCA plot. 1) and 2) represent reduced- and elevated-metabolite levels, respectively, in diapause-destined pupal brain. The samples within a treatment group were circumscribed spatially by the dashed line. The relative ratio is the ratio of metabolite peak area to internal standard (sucrose) peak area. All bars represent the mean ± S.D. from four repeats. *, *p*<0.05; **, *p*<0.01 (determined by one-way ANOVA). NP, nondiapause pupa; DP, diapause-destined pupa. Arabic number (1, 4, and 10) represent the days after pupation.

Diapause-destined pupae will enter diapause within 10 days after pupation [18]. Therefore, we investigated changes in the metabolite levels in the hemolymph and brains of the nondiapause- and diapause-destined individuals within 10 days after pupation. The first principal component (PC1) and second principal component (PC2) accounted for 44.0% and 18.9% of total variation in hemolymph PCA plot (Figure 1A); PC1 and PC2 accounted for 52.2% and 11.1% of total variation in brain PCA plot (Figure 2A). A clear separation of among groups found on PC1 of the PCA analysis showed that different physiological states in the hemolymph and brains are found in the nondiapause- and diapause-destined individuals on day 4 (Figure 1A and 2A). This finding indicates that the metabolic profiles of diapause- and nondiapause-destined pupae begin to differentiate on day 4.

In a previous study, we investigated changes of sugar (trehalose and glucose) and TCA cycle-related intermediates (pyruvate, isocitrate, fumarate, succinate, and malate) in hemolymph and brains of diapause- and nondiapause-destined pupae during diapause initiation, maintenance and termination. At diapause initiation, we showed that 1) the levels of trehalose, succinate, and malate increase in the hemolymph, but glucose and pyruvate, decrease; 2) the levels of trehalose and glucose increase in the brain, but pyruvate, isocitrate, and succinate decrease [19]. In this study, statistical comparisons between the hemolymph from the nondiapause- and diapause-destined individuals revealed that 14 metabolite levels significantly changed at least one of the groups, including 11 amino acids (*β*-alanine, glutamate, isoleucine, leucine, methionine, phenylalanine, proline, serine, threonine, tyrosine, and valine), one polyol (myo-inositol), one small molecule (phosphoric acid), and one other metabolite (butanoic acid). Of these metabolites, the levels of four (methionine, tyrosine, glutamate, and phenylalanine) were significantly lower in the diapause-destined pupae compared with the nondiapause-destined pupae. The tyrosine level in day 10 diapause-destined pupae is only half as much as its nondiapause-destined counterparts (Figure 1B-1). Ten metabolite levels were significantly higher, including *β*-alanine, isoleucine, leucine, proline, serine, threonine, valine, myo-inositol, phosphoric acid, and butanoic acid. For example, the levels of *β*-alanine and phosphoric acid in diapause-destined pupae are twice as much as its nondiapause-destined counterparts (Figure 1B-2).

Furthermore, we found 10 altered metabolite levels in the brains of the diapause-destined individuals, which included seven amino acids (alanine, aspartate, N-acetyl-glutamine, isoleucine, leucine, tyrosine, and valine), one small molecule (phosphoric acid), and two other metabolites (D-gluconic acid and pantothenic acid). All of these metabolite levels were reduced in the diapause-destined pupal brain (Figure 2B-1) except for one metabolite, aspartate, which had an elevated level during diapause initiation (Figure 2B-2).

### Changes in metabolite levels during diapause maintenance

To compare the changes in the metabolite levels between the nondiapause and diapausing individuals, day 3 nondiapause pupae (NP3) and day 21 diapausing pupae (DP21) were evaluated. First, all of the metabolites identified in the NP3 and DP21 pupae were subjected to PCA analysis. The hemolymph PCA score plot showed that the PC1 and PC2 accounted for 65.4% and 13.9% of total variability, respectively (Figure S1A). The brain PCA score plot showed that the PC1 and PC2 accounted for 55.9% and 16.8%, respectively (Figure S1B). The PCA results showed different groups separated along PC1, which indicates that there was a significant difference in the physiological state in the brain and hemolymph of the nondiapause and diapausing individuals.

**Figure 3:**
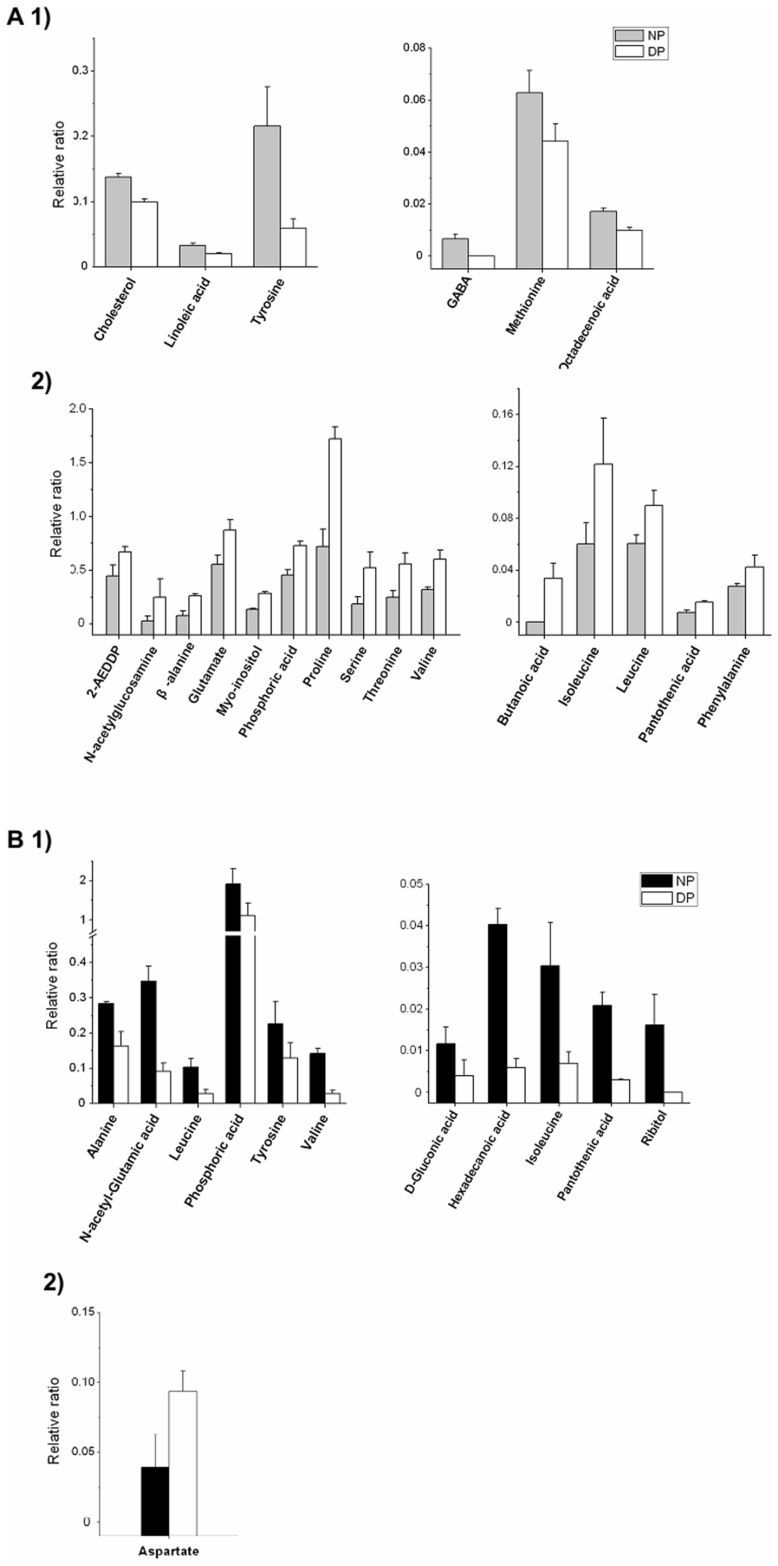
Metabolite contents in the hemolymph and brain during diapausing. Metabolite content in the hemolymph (A) and brain (B) of nondiapause pupae (NP) and diapausing pupae (DP). 1) and 2)represent the significantly reduced-and elevated-metabolite levels, respectively, in the diapause pupal hemolymph or brain (*p*<0.05; determined by one-way ANOVA). The relative ratio is the ratio of the metabolite peak area to the internal standard (sucrose) peak area. All of the bars represent the mean ± S.D. from four replicates.

As previous report, we showed that the levels of trehalose and isocitrate increase in the hemolymph, but glucose, pyruvate, malate, and fumarate decrease [19]. In this study, there were 21 altered metabolite levels in the hemolymph. 1) The levels of six metabolites, cholesterol, GABA, linoleic acid, octadecenoic acid, methionine, and tyrosine, were reduced (Figure 3A-1); and 2) the levels of 15 metabolites, 2-aminoethyl dihydrogen phosphate (2-AEDDP), *β*-alanine, butanoic acid, glutamate, isoleucine, leucine, myo-inositol, pantothenic acid, phenylalanine, phosphoric acid, proline, serine, threonine, valine, and N-acetylglucosamine, were elevated. For example, the levels of isoleucine, proline, and serine in diapausing pupae are twice as much as it in nondiapause pupae (Figure 3A-2).

Additionally, 12 metabolite levels were significantly altered in the brains of the diapause pupae. Of these metabolites, 11 metabolite levels were lower in the diapause pupal brain (alanine, D-gluconic acid, N-acetyl-glutamine, hexadecanoic acid, isoleucine, leucine, pantothenic acid, phosphoric acid, ribitol, tyrosine, and valine). The hexadecanoic acid level in diapausing pupae is approximate one-fourth as much as it in nondiapause pupae (Figure 3B-1). Only the level of one metabolite, aspartate, was higher in the diapausing pupal brain (Figure 3B-2).

### Changes in metabolite levels during diapause termination

**Figure 4:**
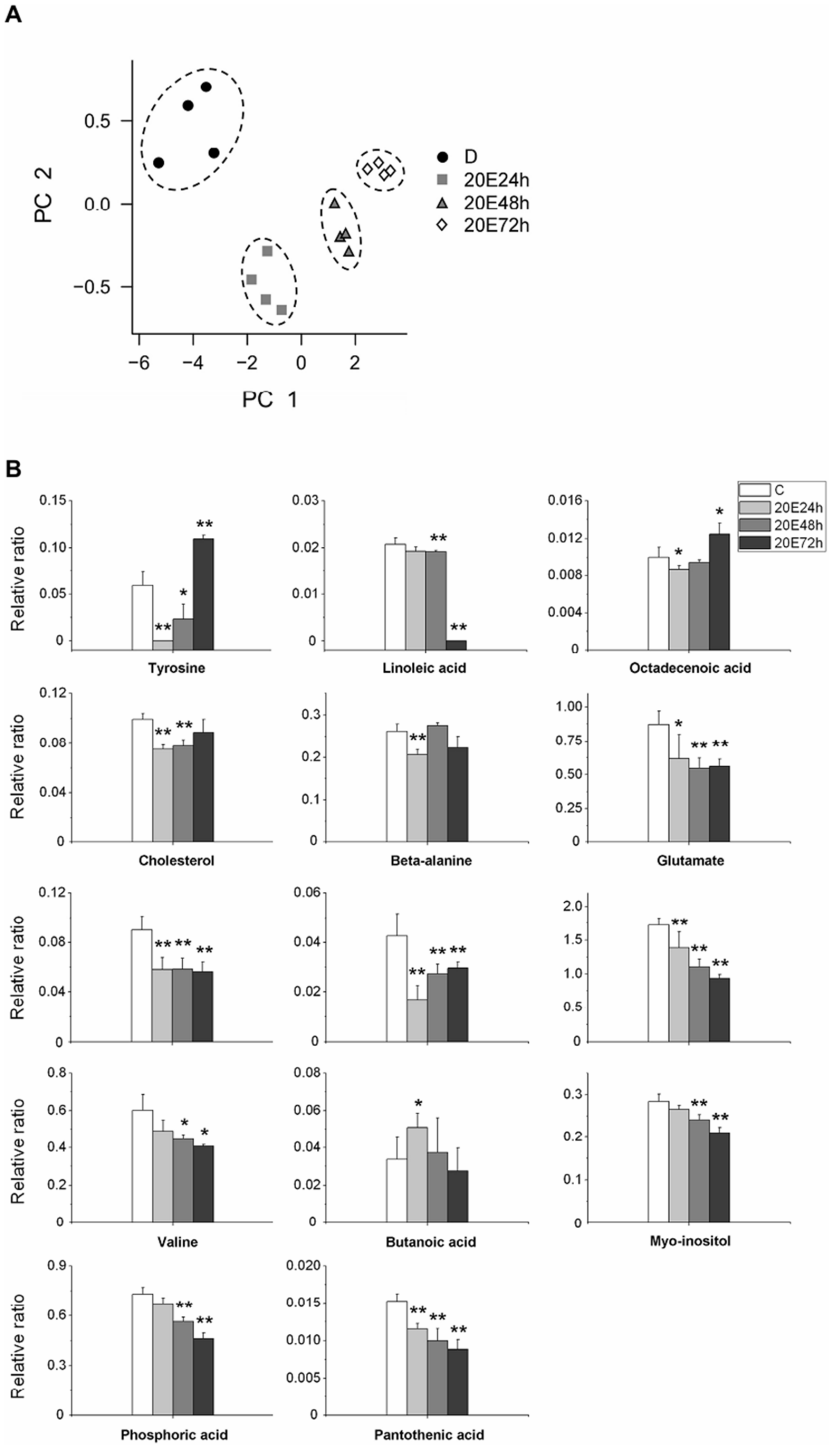
Metabolite content in the diapausing pupal hemolymph during diapause termination resulting from injection of 20-Hydroxyecdysone (20E). Pupae were incubated at 25 °C after injection. The metabolites in the hemolymph were detected at 24, 48, and 72 h after injection. C represents the diapausing pupae not given the 20E injection and used as a control. (A) The PCA analysis for the metabolites in the hemolymph after injection. PC1 containing valine, leucine, proline, and phosphoric acid accounted for 39.1% of total variation, and PC2 containing butanoic acid, phenylalanine, tyrosine, and cholesterol accounted for 21.2% of total variation in brain PCA plot. The samples within a treatment group were circumscribed spatially by the dashed line. (B) Altered metabolite levels during diapause termination. The relative ratio is the ratio of the metabolite peak area to the internal standard (sucrose) peak area. All of the bars represent the mean ± S.D. from four replicates. *, *p*<0.05; **, *p*<0.01 (determined by one-way ANOVA).

Finally, 20E was injected into diapausing pupae to investigate diapause termination, and changes in metabolite levels were evaluated at 24, 48, and 72 h after injection. The PC1 and PC2 accounted for 39.1% and 21.2% of total variability, respectively. The PCA score plots showed that there were clear changes in the metabolite levels 24 h after injection (Figure 4A).

As described in a previous report, we showed that trehalose and isocitrate reduce in the hemolymph at diapause termination, but glucose, pyruvate, succinate, and malate increase [19]. Here, there were 14 altered metabolite levels in the hemolymph after 20E injection. A dramatic decrease in the levels of 13 metabolites (glutamate, leucine, phenylalanine, *β*-alanine, proline, valine, cholesterol, octadecenoic acid, tyrosine, myo-inositol, pantothenic acid, linoleic acid, and phosphoric acid) was observed at 24 h after injection. Only the butanoic acid level demonstrated a slight increase. Of the 13 metabolites with reduced levels, the levels of 11 metabolites continued to decrease until 72 h after injection. At 72 h after injection, only the octadecenoic acid and tyrosine levels demonstrated significant increases (Figure 4B).

## Discussion

Insects cease to develop and their metabolic activity is reduced when they enter diapause [3]. In this study, we systematically investigated the changes in the metabolite levels in *H. armigera* pupae during diapause initiation, maintenance, and termination to better understand the molecular mechanisms underlying diapause. A total of 55 metabolites in the pupal hemolymph and 52 metabolites in the pupal brain were identified. Except for glucose, trehalose, pyruvate, and TCA-related metabolites (fumarate, succinate, malate, and isocitrate), which have been reported in a previous study [19], a total of 21 and 12 altered metabolite levels were detected in the hemolymph and brain, respectively.

### Changes in metabolite levels during diapause initiation

#### 1. Carbohydrate metabolism

At diapause initiation, trehalose, succinate, and malate increased in hemolymph of diapause-destined pupa, but glucose and pyruvate decreased, suggesting that low levels of glucose and pyruvate control brain metabolic activity to induce diapause entry [19]. In the brain of diapause-destined pupa, trehalose and glucose increased, but pyruvate and isocitrate decreased. This result suggests that trehalose and glucose are used as energy resource, and low levels of pyruvate and isocitrate control TCA cycle activity. Therefore, the carbohydrates play a key role in developmental arrest.

#### 2. Amino acid metabolism

The amino acid profile was altered in the hemolymph of diapause pupae. The levels of four different amino acids (methionine, tyrosine, glutamate, and phenylalanine) were lower, and the levels of seven different amino acids (*β*-alanine, isoleucine, leucine, proline, serine, threonine, and valine) were higher. These amino acids are closely correlated with carbohydrate metabolism, especially the TCA cycle.

Leucine, isoleucine, *β*-alanine, and valine can be synthesized from pyruvate [20]. In this study, we found that these amino acids in hemolymph gradually increased from day 1 to day 10 after pupation, whereas pyruvate was decreased. It implies that the decreased pyruvate level is likely related to high amino acid level. Our results are consistent with previous studies that there is a higher leucine level in *S. crassipalpis* diapausing pupae [16] and *B. mori* diapausing embryos [21]. Furthermore, our results are also consistent with other previous studies showing that the alanine level is higher in *Mamestra brassicae* and *Enosima leucotaeniella* diapause individuals [22], [23]. Therefore, an increase in these four amino acids is likely correlated with altered pyruvate levels in the hemolymph, and low pyruvate level is necessary for inducing pupal diapause through repressing the TCA cycle in the brain [19].

The levels of proline and serine, which can be used to synthesize pyruvate, increased in the hemolymph, suggesting that these two amino acids may serve as a source of pyruvate in the *H. armigera* hemolymph during the diapause phase. Thus, higher levels of these two amino acids suggest that there is an increased reserve of pyruvate. This result is consistent with previous studies showing that proline accumulates and serves as an energy source in *B. mori* diapause eggs [24] and the diapausing drosophilid fly, *Chymomyza costata* [25].

It has been well established that methionine is the first amino acid in protein synthesis, that glutamate plays a crucial role in the synthesis and reactivity of the protein porphyrin inosinic acid and that glutathione, tyrosine and phenylalanine are involved in the synthesis of melanin and neurotransmitters [20]. Thus, a decrease in these amino acids in the hemolymph may down-regulate protein synthesis and metabolic activity in the brain.

#### 3. Polyol and fatty acid metabolism

An accumulation of cryoprotectants affects the supercooling point and enhances the resistance against low temperatures, which may influence an insect's survival rate. We observed higher levels of trehalose and sorbitol, which are consistent with previous reports [5], [19], as well as a higher level of myo-inositol during diapause initiation. Higher levels of trehalose, sorbitol and myo-inositol have also been observed in diapausing pistachio fruit hull borer (*Arimania comaroffi*) pupae during overwintering [26]. Although glycerol is a well-known cryoprotectant that has been found in other diapausing insects [6], [16], [27], [28], we did not detect a higher glycerol level in the hemolymph and brain of diapausing *H. armigera*, which is consistent with a previous study [5]. This finding suggests that *H. armigera* is not a glycerol-dependent diapause species and predominantly depends on trehalose, sorbitol and myo-inositol for cryoprotection. Furthermore, these cryoprotectants are synthesized during glycolysis, and an accumulation of these cryoprotectants implies that energy metabolism is reduced and affects the brain metabolic activity.

#### 4. Other metabolites

Pantothenic acid (vitamin B5) is an essential nutrient and is used to synthesize co-enzyme A (CoA) in animals [29]. CoA is a vital metabolic intermediate that plays important roles in glucose metabolism and the biosynthesis of fatty acids and cholesterol. The level of pantothenic acid is reduced in the diapause-destined pupal brain, which suggests that CoA synthesis is reduced, and a low level of CoA may relate to lower TCA cycle activity and lower metabolic activity.

In a previous study, we found that the phosphorylated protein level in the brain is reduced during diapause initiation [18]. In this study, we found that the phosphoric acid level increased in the diapause pupal hemolymph and decreased in the diapause pupal brain. This result is consistent with a previous study [30] and suggests that phosphoric acid may be involved in the change in the phosphorylated protein level observed in the brain, which would result in a decrease in enzyme activity and regulate specific signaling pathway. The higher phosphoric acid level in the hemolymph may serve as a resource for pupal-adult development after diapause termination.

During diapause initiation, most of the metabolite levels increased in the hemolymph of diapause-destined pupae, whereas all of the metabolite levels, except for aspartate, decreased in the brain. An increase in metabolites in the hemolymph may serve as a resource for the long diapause phase and for pupal-adult development after diapause termination. The down-regulation of metabolites in the brain may serve to reduce brain activity, as previous studies have shown that there is low brain activity in diapause-destined *H. armigera* individuals [19], [31].

### Changes in metabolite levels during diapause maintenance

Except for sugar and TCA-related metabolites, the levels of six metabolites decreased and the levels of 15 metabolites increased in the hemolymph of diapausing pupae. The levels of 12 metabolites decreased and only the aspartate level increased in the brains of the diapausing pupae.

#### 1. Carbohydrate metabolism

At diapause maintenance, glucose, pyruvate, fumarate, and malate decreased in diapause-destined pupal hemolymph, but trehalose and isocitrare increased [19]. This result suggests that trehalose is main energy resource for maintaining diapause, and low levels of glucose and pyruvate in the hemolymph act as pivotal limiting factors to inhibit TCA cycle activity of the brain.

#### 2. Amino acid metabolism

In the diapausing pupal hemolymph, two amino acid levels (methionine and tyrosine) decreased, and nine amino acid levels (glutamate, *β*-alanine, proline, serine, threonine, valine, isoleucine, leucine, and phenylalanine) increased. As previously mentioned, the reduced levels of methionine and tyrosine in the hemolymph may be used as limiting factors to repress protein synthesis. The increased amino acid levels in the diapause pupal hemolymph may serve as a resource for pupal-adult development after diapause termination.

In the diapausing pupal brain, five amino acid levels (alanine, leucine, tyrosine, valine, and isoleucine) decreased, and only the aspartate level increased. The five amino acids with reduced levels are closely correlated with pyruvate, CoA, and succinate, which serve as TCA intermediates and suggest that low levels of these amino acids may be the limiting factor that reduces brain activity. Interestingly, a high level of aspartate was detected in the brain. Aspartate is the amino acid most closely associated with carbohydrate metabolism because aspartate can be converted to oxalate, which can be used to synthesize citric acid for the TCA cycle. In addition, other amino acids can be synthesized from aspartate, including four essential amino acids: methionine, threonine, lysine, and isoleucine. Therefore, aspartate can be considered an energy source and an amino acid resource for brain metabolism during a long diapause phase.

#### 2. Polyol and fatty acid metabolism

The cryoprotectant myo-inositol level was higher in the brain during diapause, and as previously mentioned, myo-inositol may protect the brain from harsh environments. Fatty acids are the primary energy source, and we observed reduced levels of two fatty acids, linoleic acid and octadecenoic acid, in the diapause pupal hemolymph. Trehalose is the predominant sugar found in insect blood. The reduced fatty acid levels may be caused by trehalose synthesis, which can be easily used during the diapause phase. However, this method used does not allow detailed analysis of changes in fatty acids composition associated with diapause. Therefore, the function of these fatty acids with altered levels in diapause insects should be investigated in future studies.

#### 3. Other metabolites

Both the pantothenic acid and phosphoric acid levels increased in the diapausing pupal hemolymph and decreased in the pupal brain. This finding suggests that pantothenic acid and phosphoric acid serve are source function and accumulate in the hemolymph and that the low pantothenic acid and phosphoric acid levels in the brain imply a reduced metabolic activity. It has also been shown that the pantothenic acid level increases in ground squirrel plasma during the hibernating period [32]. These findings suggest that pantothenic acid may play a similar role in insects and animals demonstrating low metabolic activity.

### Changes in metabolite levels during diapause termination

At diapause termination, trehalose and isocitrate decreased in hemolymph of diapause-destined pupa, but glucose, pyruvate, succinate, and malate increased [19]. This result suggests that the energy reserves are heavily used and the brain metabolic activity increases to promote pupal-adult development.

When 20E was injected into the diapausing pupae to terminate diapause, 14 metabolite levels in the hemolymph were altered. The levels of 13 metabolites (glutamate, leucine, phenylalanine, *β*-alanine, proline, valine, cholesterol, octadecenoic acid, tyrosine, myo-inositol, pantothenic acid, linoleic acid, and phosphoric acid) were lower at 24 h after injection. Only the butanoic acid level was slightly increased. The glutamate, alanine, and proline levels decreased significantly, and these results suggest that there is an increase in the pyruvate level for TCA cycle activity, which would promote pupal-adult development [19]. Threonine and valine can be used to synthesize succinate for the TCA cycle. All of the metabolite levels decreased during diapause termination, except for the butanoic acid level, which suggests that these metabolites may be involved in the metabolic activity of the tissues and organs for resuming development.

## Conclusions

**Figure 5:**
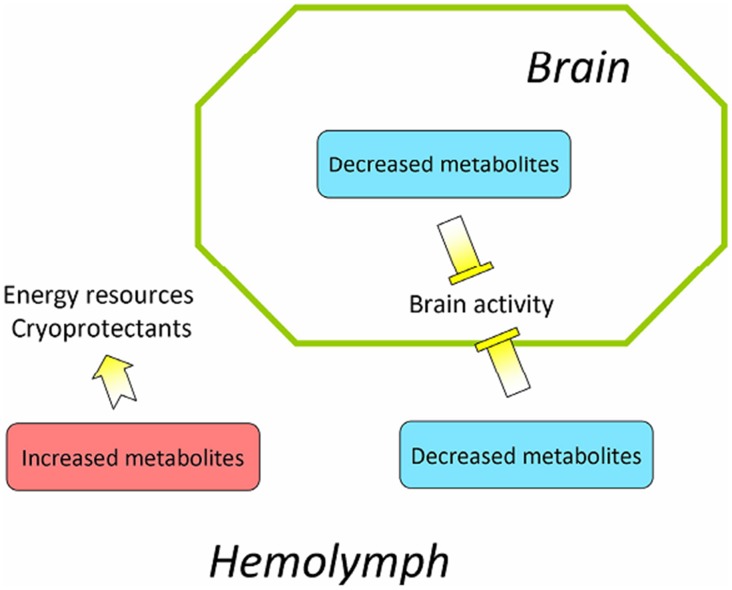
Possible mechanisms underlying insect pupal diapause. The increased metabolite levels in the hemolymph serve as energy sources and cryoprotectants for use during a long pupal diapause phase to survive harsh environmental conditions and to sustain pupa-adult development after diapause termination. The decreased metabolite levels in the hemolymph and brain are limiting factors that contribute to repressing brain activity.

The GC-MS analyses of diapause *H. armigera* pupal hemolymph and brain successfully identified a cluster of metabolites that are involved in carbohydrate metabolism, amino acid metabolism, as well as polyol and fatty acid metabolism during the initiation, maintenance, and termination of pupal diapause. 1) At diapause initiation, the metabolite levels gradually increase in the pupal hemolymph and serve as a resource for pupa-adult development after diapause termination; the metabolite levels in the brain are reduced and correlated with low metabolic activity in the brain during diapause entry. 2) During diapause maintenance, the number of metabolites with increased levels in the pupal hemolymph is greater than the number of metabolites with decreased levels. These increased metabolites will serve as an energy source and as metabolic intermediates for maintaining the activity of the brain and other organs during a long diapause period and for pupal-adult development after diapause termination. The reduced metabolites in the diapause pupal hemolymph may contribute in the down-regulation of brain activity that helps to maintain the diapause state. In the diapause pupal brain, the number of reduced metabolites is far greater than the number of increased metabolites, which suggests that the low metabolite levels are crucial for low brain activity. 3) During diapause termination, most metabolite levels rapidly decrease in the hemolymph in response to the 20E signal and serve as energy and metabolic sources that promote pupa-adult development. Combing this study with previous reports, suggest that not only metabolites are energy resources and cryoprotectants, but also can serve as regulators [17], [19], [30]. Based on the results from these studies, a schematic diagram illustrating a proposed general mechanism by which metabolites regulate insect diapause is presented (Figure 5).

## Supporting Information

Figure S1 **Principal component analysis (PCA) score plots for metabolites during diapausing.** The metabolites of hemolymph (A) and brain (B) during diapause maintenance. PC1 containing valine, leucine, threonine, tyrosine, linoleic acid, octadecenoic acid and phosphoric acid, etc. accounted for 65.4% of total variation, and PC2 containing glycine and D-gluconic acid accounted for 13.9% of total variation in hemolymph PCA plot (A). PC1 containing valine, alanine, leucine, isoleucine, and phosphoric acid accounted for 55.9% of total variation, and PC2 containing proline, phenylalanine, and inositol accounted for 16.8% of total variation in brain PCA plot (B). NP, nondiapause pupa; DP, diapausing pupa.
(TIF)
