# Supplementary figures and images for: Global Metabolomic Analyses of the Hemolymph and Brain during the Initiation, Maintenance, and Termination of Pupal Diapause in the Cotton Bollworm, Helicoverpa armigera
Source: PLoS One. 2014 Jun 13;9(6):e99948. doi: 10.1371/journal.pone.0099948 (PMC4057385; doi:10.1371/journal.pone.0099948)

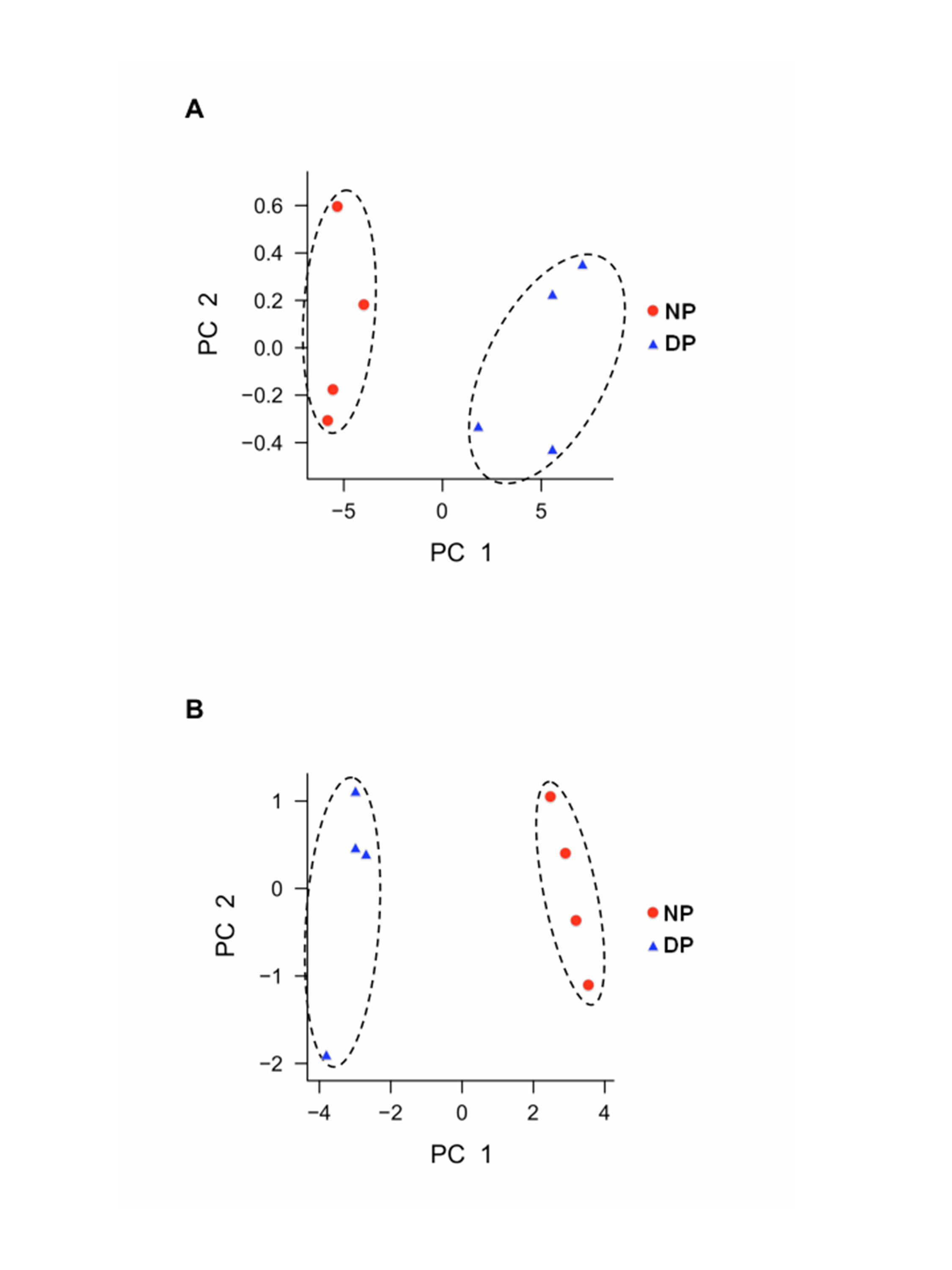

Supplement: Figure S1 — Principal component analysis (PCA) score plots for metabolites during diapausing. The metabolites of hemolymph (A) and brain (B) during diapause maintenance. PC1 containing valine, leucine, threonine, tyrosine, linoleic acid, octadecenoic acid and phosphoric acid, etc. accounted for 65.4% of total variation, and PC2 containing glycine and D-gluconic acid accounted for 13.9% of total variation in hemolymph PCA plot (A). PC1 containing valine, alanine, leucine, isoleucine, and phosphoric acid accounted for 55.9% of total variation, and PC2 containing proline, phenylalanine, and inositol accounted for 16.8% of total variation in brain PCA plot (B). NP, nondiapause pupa; DP, diapausing pupa. (TIF) [file pone.0099948.s001.tif]
